# eFisioTrack: A Telerehabilitation Environment Based on Motion Recognition Using Accelerometry

**DOI:** 10.1155/2014/495391

**Published:** 2014-01-12

**Authors:** Daniel Ruiz-Fernandez, Oscar Marín-Alonso, Antonio Soriano-Paya, Joaquin D. García-Pérez

**Affiliations:** ^1^Department of Computer Technology, University of Alicante, Carretera San Vicente del Raspeig s/n, San Vicente del Raspeig, 03690 Alicante, Spain; ^2^Bio-inspired Engineering and Health Computing R.G. (IBIS), University of Alicante, Carretera San Vicente del Raspeig s/n, San Vicente del Raspeig, 03690 Alicante, Spain

## Abstract

The growing demand for physical rehabilitation processes can result in the rising of costs and waiting lists, becoming a threat to healthcare services' sustainability. Telerehabilitation solutions can help in this issue by discharging patients from points of care while improving their adherence to treatment. Sensing devices are used to collect data so that the physiotherapists can monitor and evaluate the patients' activity in the scheduled sessions. This paper presents a software platform that aims to meet the needs of the rehabilitation experts and the patients along a physical rehabilitation plan, allowing its use in outpatient scenarios. It is meant to be low-cost and easy-to-use, improving patients and experts experience. We show the satisfactory results already obtained from its use, in terms of the accuracy evaluating the exercises, and the degree of users' acceptance. We conclude that this platform is suitable and technically feasible to carry out rehabilitation plans outside the point of care.

## 1. Introduction

**Figure 1:**
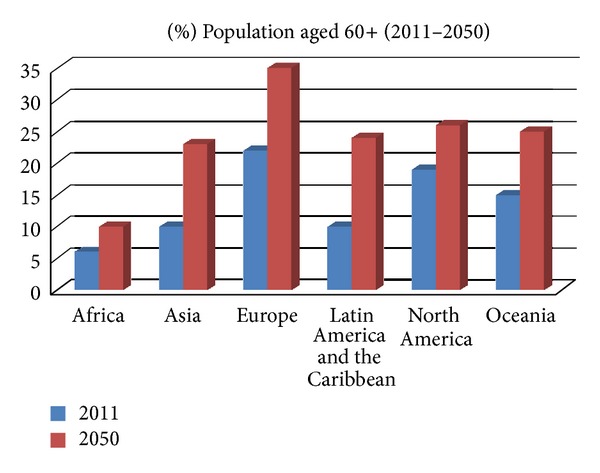
Population aging prospects of the United Nations' Department of Economic and Social Affairs. Available online: http://esa.un.org/unpd/wpp/index.htm (last accessed on 10 March 2013).

Nowadays there is an ever-growing demand for rehabilitation processes within public and private healthcare services. In 2011 the World Health Organization estimated that one billion people worldwide were in need of rehabilitation treatments [1]. What is more, this increasing demand is expected to continue in time as the population that aged over 60 will grow (Figure 1). This is a worldwide matter that in developed countries reaches its maximum due to better welfare and health conditions.

Aging is recognised as one of the main causes of the need for any kind of rehabilitation treatment, and its importance lies in the fact that it is unavoidable. Focusing on physical rehabilitation, besides ageing, there are other prominent causes for these therapies: musculoskeletal injuries, illnesses that affect the locomotor system, and postsurgery scenarios. All these situations cause the loss of physical abilities, having a clear impact on patients' quality of life.

Physical rehabilitation plans involve a set of different therapies that aim to return patients to a healthy condition allowing them to recover a normal way of living. The objectives and the design of these plans vary depending on temporary aspects of the disabilities. In case of permanent disabilities, rehabilitation will probably last until the end of the patient's life, having as unique goal the maintenance of his self-sufficiency, or just decelerate the loss of physical abilities. If the disabilities are temporary, the objective of rehabilitation therapies is to help patients to regain as much self-sufficiency and lost skills as possible, in the minimum time, in order to live a normal life.

As it is easy to deduce, physical rehabilitation processes are highly cost-consuming, becoming a threat to the sustainability of healthcare services. Rehabilitation plans are required to be as efficient as possible to make them affordable to funding institutions. In these plans, efficiency depends on its logistics, organizational factors, and healthcare policies. In addition to this, patients' attitudes and behaviour have a great impact on this issue due to the concept of patients' treatment adherence. Treatment adherence is related to the degree in which patients follow the experts' guidance and fulfill the scheduled clinical activities prescribed to them [2]. The lack of adherence within physical rehabilitation programmes has been identified as one of the most important reasons for treatment failure in pathologies of the locomotor system [3, 4]. There are studies showing that situations of poor adherence to treatment can result in longer recovery periods, growing waiting lists, or even new injuries caused by a wrong performance of the prescribed exercises [5, 6]. We can state that the lack of adherence physical rehabilitation treatments directly affects to the quality of rehabilitation services and the patients' quality of life.

**Figure 2:**
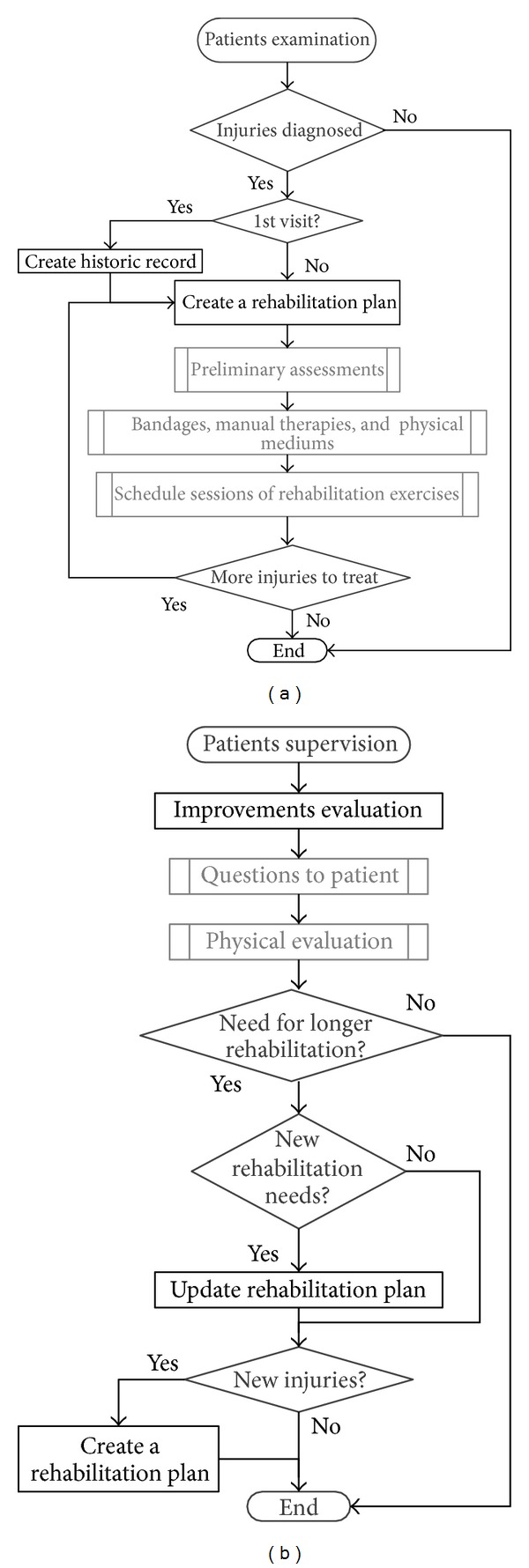
Flow diagram of rehabilitation attention processes. On the left, patient's 1st examination and on the right, patient's improvements supervision.

Patient's attitude towards rehabilitation treatments can be influenced by several factors, which are related to him, his relation with the rehabilitation experts, and organizational issues of the rehabilitation services. An example of this is the importance of the scenarios in which the rehabilitation processes take place. We can distinguish between two main situations:

- inpatient scenario, in which the patient goes to a point of care in scheduled sessions and performs the exercises partially supervised by experts, since each physiotherapist attends many patients at a time. In (Figure 2) we can see flow diagrams of two common rehabilitation activities: first examination to evaluate patient's condition and later supervision of the improvements made by the patient through a rehabilitation plan;
- outpatient scenario, in this case after the prescription of a rehabilitation plan, the patient performs, usually at home, the exercises under no supervision. Experts check his evolution through regular meetings at the point of care.

Inpatient scenarios require higher personnel cost but allow a more regular communication between patients and physiotherapists. On the contrary, in outpatient situations this communication is almost nonexistent. So it is highly likely that the patient does not remember clearly the expert's instructions to perform the exercises in his day-to-day rehabilitation sessions. Even more, as they perform them without supervision, they will not receive any feedback about the correctness of their performance, until the next scheduled visit to the physiotherapist office.

In their study, Jack et al. observe the difference between adherence to treatment of patients involved in inpatient and outpatient scenarios, respectively [7]. Patients' motivation and adherence to treatment tend to decrease as they go through the rehabilitation plan in outpatient scenarios. The study from Sniehotta et al. shows that motivation and involvement of patients are reinforced by an effective communication with the clinical experts, making the odds of patient adherence 2.16 higher [8]. Situations of low motivation and lack of involvement can result in low self-efficacy situations. All of them are important barriers to treatment's adherence [9].

Within the field of information and communication technologies (ICT) we can find suitable solutions to face the previously described issues related to physical rehabilitation processes. After meeting with physiotherapists and reviewing related works, we decided to design a system to allow patients to perform rehabilitation exercises in outpatient scenarios but assuring the real-time communication between them and the experts at the point of care. In short, we aim to reduce the workload of healthcare services, making them more efficient, while eliminating the gap between patients and experts improving their experiences by using a low-cost and easy-to-handle platform.

To achieve those goals our system should monitor the patients' performance of the prescribed exercises, evaluate its correctness, and securely store these data. Furthermore, patients and physiotherapists should have access at any time (24 × 7 × 365) to all the data related to the rehabilitation plan, to obtain useful feedback. In this work we described the objectives of our project, its final design, and the test and validation processes carried out to show its feasibility to be used for telerehabilitation purposes.

This paper is organized as follows. In Section 2, we show previous approaches from other researchers to the issues we face. In Section 3 we show our platform's specification describing the software and hardware components and the offered services to physiotherapists and patients. Next, in Section 4 we show the platform in use, describing the evaluation procedures that have been carried out to test its validity. Finally, in Section 5 we show conclusions obtained after the design, implementation, and validation stages, including future works to be developed from now on.

## 2. Related Works

The objective of improving the quality of health care and the functioning of healthcare services has led to many cases of use of ICT in the medical field. Our work is connected with the concept of telemedicine, which aims to offer ubiquitous healthcare services to patients. Within this concept, we can talk about telerehabilitation as process in which a patient performs tasks related to follow a rehabilitation plan, of any kind, outside a healthcare centre.

Telerehabilitation proposals vary in their design since they are focused on different aspects of the rehabilitation process. These variations depend on the targeted user (patient or physiotherapist), their purpose (to test the use of new technologies, to improve the attention in outpatient situations, and so on), the disability and part of the body to be treated, and the environment in which the solution will be used.

There is a prominent trend of works that use virtual reality and robotics to offer to patients innovative ways to perform prescribed exercises. Some sensing devices are used to offer the virtual reality feeling and to record useful data about patient's state and behaviour [10, 11]. These proposals are often showed as games where the patients are required to make certain gestures to interact with the game device. Virtual reality-based projects are mainly focused on rehabilitation in poststroke or cerebral damage situations [12–14], while those that use robotics are aimed to rehabilitation of musuloskeletal disabilities [15, 16]. Nonetheless, these works seemed to be focused on testing the feasibility of these new ways of rehabilitation, and they do not pay much attention to user's feedback, the communication between patients and experts, and how to evaluate the progresses made by the patients.

In a ubiquitous scenario patients should be monitored as they perform the rehabilitation exercises. Afterwards, the obtained data should be at physiotherapist disposal to be evaluated or examined. Motion tracking techniques are useful to do this task. A work by Zhou and Hu shows examples of the use of multiple tracking systems for rehabilitation purposes, depending on the sensing devices and information processing algorithms used [17].

Inertial sensors have been extensively used for human motion tracking and recognition because of their low cost, simplicity, and availability. These sensors have been applied to recognize and measure human activities [18, 19] and to track and evaluate certain gestures performance [20]. Both tasks are useful in telerehabilitation scenarios.

Inertial sensors are often combined with other sensors, inertial or not. This is done to avoid some disadvantages of these sensors like drift when estimating 3D coordinates or noise in the gathered data, due to sensor rotations, displacements, or other perturbations [21–23]. The fusion of sensors helps to achieve more robust and accurate tracking systems. On the other hand, these systems have to deal with complex tasks like the fusion and processing of motion data. Furthermore, they are also intrusive and uncomfortable to wear due to the need of placing several sensors on the user's body.

Taking into account the need of a low-cost system and the patient's autonomy and comfort, we tested the possibility of using one single inertial sensor. We choose a gamepad manufactured by Nintendo, the Wii Remote©, that includes a three-axis accelerometer [24]. Wii Remote© has been used for general-purpose gesture recognition [25, 26] and in few rehabilitation projects as well [27–29]. In these projects it is used as tool to collect motion data, but it is not integrated in a wider system to support rehabilitation processes' needs.

After reviewing previous works we had insight of several issues that should be considered to design a low-cost, easy-to-use, and able-to-generalize rehabilitation system. Many of them are focused only on rehabilitating concrete parts of the body [30, 31], or the consequences of certain conditions like stroke. Due to its complexity and the use of multiple devices, their design is sometimes intrusive making some of them hardly suitable for domiciliary scenarios and affecting patients' motivation. In addition, this complexity may affect to cost, making them not affordable for healthcare services. Finally, it has been proved that issues related to users should be taken into account [32] when designing a telerehabilitation solution, moreover, if they will use the system under no supervision, as it is the case of patients in outpatient scenarios.

## 3. Platform for Physical Telerehabilitation: eFisioTrack

Our work aims to build a system whose main objective is to improve the efficiency of physical rehabilitation processes due to the benefits of outpatient scenarios. In addition, it is designed to bridge the gap between patients and physiotherapists, trying to simulate the ideal case of having an expert side by side with patients while they perform the prescribed exercises at home. At the same time, the system is intended to be helpful for physiotherapists, offering them accurate data about patients' performance allowing them to gain insight about their evolution.

### 3.1. Objectives of eFisioTrack

Primary objective of efficiency can be fulfilled through achieving other secondary ones, which can be grouped paying attention to the aspects of the rehabilitation processes they affect: technology, users (patients and physiotherapists), and rehabilitation services' functioning.

#### 3.1.1. Objectives Related to Patients and Experts

The system must give to patients and experts suitable mechanisms to interact with each other as if they were one in front of each other. In the case of patients, at the time they perform their exercises they should have at their disposal all information about the schedule of the rehabilitation plan, a full description about how to perform each exercise, and real-time feedback about their performance. For experts the system should gather information about the fulfillment of the plan and the correctness of the patients' performance. This will allow a quick and ubiquitous assessment of the patients' evolution and an update of their rehabilitation plans if needed.

System's use will help to increase patients adherence to treatment in two ways. First, since every interaction with the system is recorded, if they do not perform the exercises, if they perform them at a wrong time or in an erroneous way, the physiotherapist will instantaneously know it. Secondly, the system will avoid cases of forgetting the instructions about how to perform the exercises or the schedule of rehabilitation sessions.

#### 3.1.2. Objectives Related to Rehabilitation Services' Functioning

All the services that the system will offer must be integrated within the current rehabilitation services workflow. This means that eFisioTrack will not be a standalone application just to monitor outpatient performance of exercises. Besides, in the same environment the expert will be able to carry out all the tasks that involve dealing with patients: administrative work, agenda planning, access to patients' history, examination and prescription of complementary therapies, and so forth.

eFisioTrack is not focused on a certain joint or a part of the body. It will be able to cope with the rehabilitation of different disabilities and injuries, offering an upgradable set of exercises to do so.

#### 3.1.3. Objectives Related to Technology

The interaction mechanisms between patients and experts should be presented in a friendly environment, through comprehensive interfaces where the different options are logically disposed following common rehabilitation workflow. This is important to avoid rejection due to complexity of usage.

Achieving a low-cost system is a challenge that is taken into account at the time of the system's architecture design. This challenge is behind the election of a sensing device and the technologies used to implement both client-side and server-side software components.

To assure its scalability, the system has a modular design and each module interacts with the others through layers that abstract the offered services (access to database, schedule management, rehabilitation sessions management, etc.).

Since eFisioTrack is based on communication over the Internet some issues should be born in mind. We are dealing with clinical data which requires to set policies to preserve its privacy and integrity. Moreover, the system will offer 24 × 7 × 365 disponibility but sometimes this is impossible due to network failures. eFisioTrack can be used in these circumstances avoiding loss of information.

### 3.2. eFisioTrack's Scenario of Use

eFisioTrack will cover all the experts' needs, from the first meeting with a patient until the end of the rehabilitation process, while reducing the visits to a point of care and discharging patients from the rehabilitation services.

To achieve that, we had several meetings with physiotherapists who develop their task at a public hospital, one of them being expert on adherence to treatment issues. These meetings helped us to understand the functioning of a physical rehabilitation service and to gain insight of their needs.

**Figure 3:**
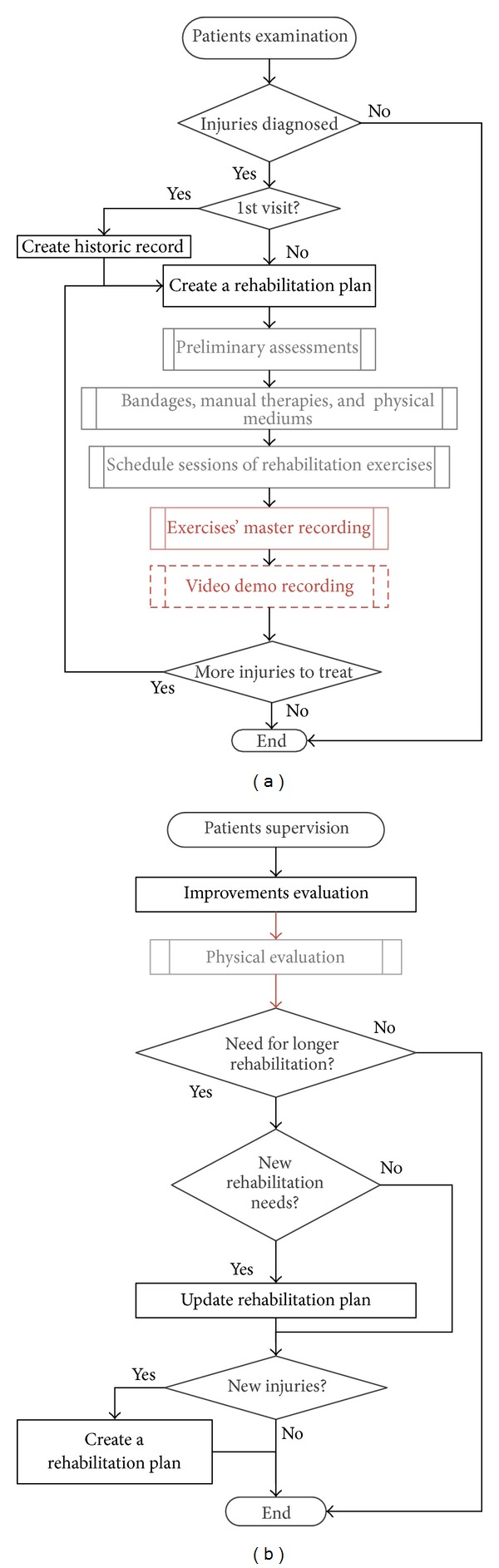
Flow diagram of rehabilitation attention processes using eFisioTrack. On the left, patient's 1st examination and on the right, patient's improvements supervision.

The use of eFisioTrack introduces little changes in experts' current procedures in order to ease the integration of the system in their workflow. In (Figure 2) we show the flow diagram of two of these procedures; now on (Figure 3) we can see how these procedures' flow is when using eFisioTrack. Changes between figures are highlighted, and the rest of processes, which stay the same, can be carried out using the system as well. It should be pointed out that the use of the system let the experts simplify patients supervision process. Now there is no need to gather information asking the patient and dealing with ambiguous answers. Experts can obtain it from the system in bigger quantity and quality.

A case of eFisioTrack use begins when a patient goes to the point of care looking for a physiotherapist's help. The physiotherapist checks his physical condition, evaluating the existence of possible injuries. According to this exploration, the expert introduces data related to patient's condition on the system and proceeds to open a new rehabilitation plan. This plan will contain a schedule of several rehabilitation exercises sessions to be done at home. Then, the expert explains to the patient how to perform each exercise, making him use the sensing device to record a motion example for each gesture. This example will be used as a realistic reference of what the patient is able to do and is useful to check the correctness of the patient's at home performances. Doing it in this way we offer a personalized treatment that fits each patient needs and abilities.

Now the patient is expected to perform the scheduled sessions at home, avoiding most of the regular visits to the healthcare centre, reducing them to the mininmum needed. In the meantime, experts can check on the system if the patient is fulfilling the rehabilitation plan and how good are the results that he is obtaining. This information is useful to modify the rehabilitation plan to fit new needs. This can be done remotely using the system or in the next appointed meeting with the patient, after a regular exploration to check his evolution.

### 3.3. Hardware Infrastructure

The telerehabilitation's environment hardware infrastructure consists of the following elements.

*(i) PC*. Computers or other general purpose devices (tablets, laptops, and so on) are suitable since eFisioTrack does not require any high performance computing resources. They are used to install the client side of the application for patients and for physiotherapists. The analysis of hardware and computational scalability made in the design phase showed that, after deploying the system, it will not be necessary to acquire any other equipment in close future. Therefore, the price of the overall system is not expected to increase, making it easier to achieve a low-cost solution.

*(ii) Sensing Device*. This device will be used to monitor the patient's performance of the exercises. It is a Wii Remote gamepad, which is a wireless and wearable low-cost motion capture device. It includes an accelerometer that gathers values of linear acceleration along three perpendicular axes (*x*, *y*, *z*). This sensor is able to measure accelerations over a range of ±3 g with 10% sensitivity and send it to the application using a BCM2042 Broadcom Bluetooth chip.

*(iii) Server*. It is where the server side of the application will be allocated. It will contain too the platform's database.

*(iv) Communication Elements.* Whatever connection to the Internet is valid, all the interactions between the client-side application and the server-side application will take place over the net.

### 3.4. Applications and Services Infrastructure

**Figure 4:**
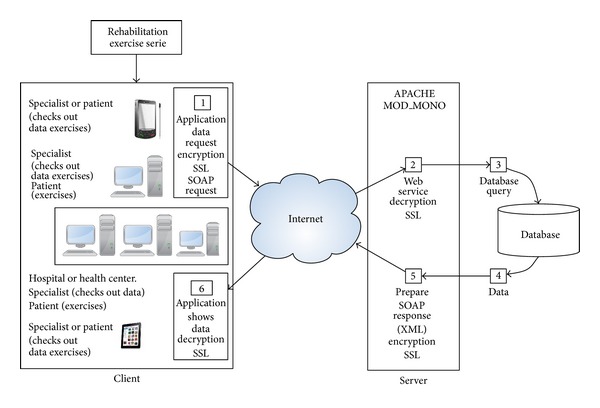
Scheme of eFisioTrack's software components and where they are used.

Software elements have been designed to fulfill the stated objectives. The set of technologies chosen and the way they interact are not casual and aim to meet those requirements: 24 × 7 × 365 connectivity, the use of low-cost motion capture devices, easy-to-use and intuitive graphical interfaces, integration of different development platforms and operating systems, and experts ubiquitous access to the data stored in the system. A diagram of the software components of eFisioTrack and the places where they are used is shown in (Figure 4).

*(i) Database*. The database is a key part of the platform since it provides the data structures for the main functionalities. Data structures have been refined since the first meeting with physiotherapists in order to offer them a complete experience like medical center consultation. The database contains data related to patients, medical records, assigned treatments, planned sessions, and so forth.

*(ii) Web Services*. The software deployed on the server follows the fourth generation of web server paradigm using XML and WSDL interfaces with SOAP-based messaging. Web services have been deployed into a 2-tier architecture (database connectivity and web server). There are three types of web services (insert, retrieve, and modify), which deal with data types defined by us to represent objects like rehabilitation sessions, exercises, and so forth.

*(iii) Connectivity*. eFisioTrack sends and receives data to and from a server, through an Internet connection. Also, an offline connectivity mode has been implemented which can be activated by the physiotherapists in the application. Once it is turned on, rehabilitation programme data are stored in the patient computer so the user does not need to log into the application again. Data remain available in the application instead of retrieving them from the server. In the case of data about the performance of rehabilitation exercises, it can be stored on a USB flash memory or on a hard drive so it can be retrieved later.

*(iv) Security and Privacy*. Since the platform environment is related to healthcare, security controls must be used to protect patient's data. The essential developed security measures are to record user actions so that every step taken in the system is known, for example, incorrect password in a login attempt. Database's structures have been designed so that database instances will be associated with neither concrete patients nor the physiotherapist as required by law. Communications are encrypted using SSL secure protocol. In addition, other precautionary measures have been taken into account such as recording every unexpected application error or filtering IP address and port of the connections made to the application server.

*(v) Technology Integration*. eFisioTrack is the result of an important effort integrating different technologies and platforms, in order to build up a sustainable and powerful system. The client side is a C# based standalone application. On the other side, the server is based on a Unix environment. The motion capture device and the application communicate using an open source library created for this purpose by Brian Peek [33].

During the development of the platform, agile software development methods have been used. Elaboration cycles have been centered in evolving functionalities over the time, as the meetings were held with physiotherapists in order to test parts of the platform. On top of that, collaboration with physiotherapist has been crucial to get the platform finished, keeping the user interface and other features such as usability and window layout design as much precise as the rehabilitation treatment needs. Throughout all the above elements, the priority has always been to use open-source elements because this helps in keeping a final affordable system budget.

*(vi) User Interfaces*. Implementation cycles have been centered in evolving functionalities over the time, as we held several meetings with the physiotherapists staff. This was crucial to design and test certain parts of the platform. An example is the different users' interfaces. Issues such as usability and window layout design were jointly examined with experts. Throughout all the above elements, the priority has always been to use open-source elements because this helps in keeping a final affordable system budget. Once we had a pilot of the system, we collect patient's opinion about the usability and usefulness perception too.

The platform has three types of users based on the specific role they play: physiotherapists, patients, and administrators. Each one of them accesses different parts of the client application; for example, the administrator adds new physiotherapist into the platform and a physiotherapist user manages all the data regarding a physiotherapy process such as add new patient and add new rehabilitation exercise. Finally a patient user can access the physiotherapy process information to perform a rehabilitation exercise or look at his scheduled elements of a specific day.

**Figure 5:**
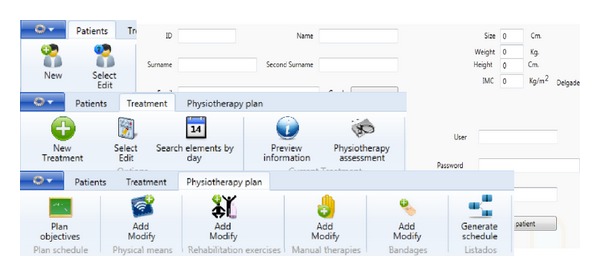
Physiotherapist options menu of the application.

When a physiotherapist logs into the application, he can choose between patients data management and physiotherapy process remote monitoring (Figure 5). In the first option, after the physiotherapist adds a new patient into the system, a new physiotherapy process can be created for that new patient. Then, the plan elements can be added into the physiotherapy process such as rehabilitation exercise session. On the other hand, data about physiotherapy remote monitoring can also be managed and edited in order to delete one element from the schedule or to add new elements at the end of the treatment if the injury has not been cured giving some advice to the patient about their realization.

When a patient accesses the application, a menu shows the elements of the active physiotherapy process. Moreover, a rehabilitation exercise can be performed with the motion capture device, and once the exercise is finished, resulting data is sent to the server. After that, the application shows the results to the user and suggests some tips to fix detected mistakes such as *remember to try staying in the same plane*. When an administrator logs into the application, a small option panel is shown that allows the administrator to add new users into the platform. It can add a physiotherapist or a new administrator since there could be the need for different administrators in the same medical center.

*(vii) Remote Monitoring*. The implementation of remote monitoring features allows the physiotherapist to totally personalize the rehabilitation program of the patient.

First, a customized movement is recorded which is considered as the master movement for a specific patient and exercise. Once the raw acceleration data is transmitted to the application, it is filtered using a three-point moving average to avoid noisy values on the signal. Then, the filtered acceleration values are the input to the motion evaluation module. This module uses the dynamic time warping (DTW) algorithm to evaluate the correctness of the patient's performed gesture [34]. DTW tries to dynamically align sequences of values, in order to estimate their similarity. In this case we compare sequences of filtered acceleration values. To do this, it uses a distance estimation function, which in our case is Euclidean distance. DTW has been used with rehabilitation purposes with satisfactory results [35]. DTW compares each exercise repetition done by the patient with the recorded master. In third place, the user knows if the exercise has been done right and the physiotherapists have more information about the performance like how many repetitions have been done right or the reasons why a repetition is wrong. Thus, the physiotherapist can quickly select proper intensity and duration values of the rehabilitation exercises.

Two clear benefits rise with this telerehabilitation scenario. First one is that with telerehabilitation we save expenses in the overall budget. The second one is contributing to the fact that the patient can perform rehabilitation exercises at home; in this way we avoid problems when traveling, since rehabilitation patients usually have problems with their mobility. Also, in the moment the physiotherapist adds a new exercise there is the option to record a personalized video which the patient can check afterwards in the moment of the exercise performance, reminding the patient how to do it properly. Furthermore, the remote monitoring has been designed using widely accepted guidelines for exercise prescription.

## 4. Evaluation

The system has been evaluated at several stages, different issues being tested at each stage. Through the design and implementation stage, each module was twice tested, first separately and once integrated in the system, jointly with the other modules. Notwithstanding this early testing, the main evaluation is made on the complete and deployable version of the system to check if it meets the stated requirements and fulfills their objectives. This process is prior to final deployment with real patients.

We designed a twofold evaluation process. On one hand, we wanted to test system's ability to monitor patient's exercises performance. We carried out a quantitative study to test eFisioTracks accuracy on doing that. On the other hand, we want to know users' opinion about the system's use: its usability, usefulness perception, friendly design, and so forth. Users took part in a qualitative study which consists of a survey of 10 simple and straight questions about their experiences.

### 4.1. Experimental Setup for Patients' Monitoring Evaluation

eFisioTrack is designed to manage any kind of rehabilitation exercises related to injuries that affect any part of the body. It includes a catalog of more than 80 different exercises mainly related to orthopedic injuries like subacromial impingement, rotator cuff tendinopathy, slap lesion, and so forth.

**Figure 6:**
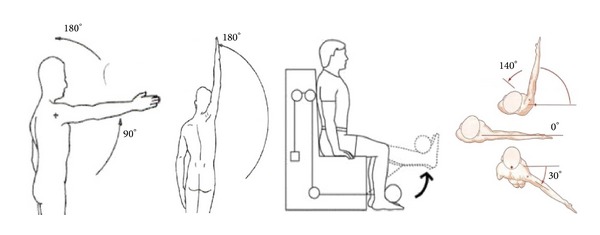
Exercises performed using eFisioTrack whose results are included in this work. From left to right: free active shoulder flexion, free active shoulder abduction, leg extension, and free horizontal shoulder adduction.

To evaluate how well eFisioTracks monitor and evaluate patients at home exercises' performance, we select 4 commonly prescribed exercises: free active shoulder flexion, free active shoulder abduction, leg extension, and free horizontal shoulder adduction (Figure 6). Each of these gestures implies a quite different motion pattern (frontal-vertical using the shoulder or the knee as motion joint, lateral-vertical, and frontal-horizontal). This will allow us to deeply test if the motion tracking algorithm is able-to-generalize, accurate, and exercise-independent.

*Setup.* The test was performed at the rehabilitation service facilities of a public hospital. Two computers and two Wii Remote gamepads were used to perform the selected exercises. Each user completes each session on different days and at different times, trying to simulate the behaviour of a real patient who will carry out the rehabilitation session whenever he likes within the scheduled day.

*Participants*. 10 volunteers took part in the tests. Since there is no a targeted user, we select people of different gender and from as widest range of age as possible. There were 6 men and 4 women, from 20–35 (5), 36–50 (3), and older than 50 (2).

*Test Development*. A physiotherapist designed a short rehabilitation plan that consisted of 4 sessions on which the testers should perform 10 times each exercise. They perform each session on different days, under the supervision of a physiotherapist. Testers deliberately perform correctly or erroneously each repetition. They even tried to trick the system performing repetitions that were almost correct but were not satisfactory enough to be considered as correct by the supervising expert. This is done to test the accuracy of the motion recognition algorithm, when comparing acceleration sequences that have different degrees of similarity. Finally we counted how many times the system's motion evaluation coincided with the expert's opinion.

*Results*. 1600 cases were considered, 800 performed correctly and 800 performed erroneously. In Table 1 we can see the obtained results. They are shown as percentages of accurate recognition cases of both correct and erroneous performed repetitions, for each exercise.

### 4.2. Evaluation of System's Perception by the Users

Apart from testing system's functionality, it is crucial to check if its use is appealing to users. We designed a qualitative study on which users are asked to fill a questionnaire of 10 questions. Questions are related to aspects of the system or its use and participants answer rating these aspects using a scale that goes from 0 to 5. A 0 rate meant “*absolutely not*,” while a 5 rate meant “*yes, absolutely*.”

In Table 2 we can see the questions included in the questionnaire and the average rate obtained in response to each question.

## 5. Conclusions

In this paper we present eFisioTrack, which is a platform that allows carrying out physical rehabilitation plans in outpatient scenarios. We developed this platform trying to offer solutions to issues like healthcare services efficiency and patients involvement in the rehabilitation processes.

eFisioTrack offers new ways to improve both physiotherapist and patient experience. It aims to reduce healthcare cost allowing performing rehabilitation plans at patient's home, discharging patients from the points of care, allowing experts to simultaneously deal with a higher number of patients, and finally shortening waiting lists. From the patients' point of view, it diminishes the discomfort reducing significantly his visits to the healthcare centre.

From the first moment, we met physiotherapists with expertise in physical rehabilitation processes. We analyse their workflow and needs, which was very useful at the time of deciding which services our system will offer. This process allowed us to avoid a later possible rejection to use the system due to not being suitable or easy to integrate in experts' routines. Other considered aspects were the cost of the system, its usability, and the simplicity of its use.

In this work we show the results from evaluating 2 key issues of the system. First, we tested how good recognizing and evaluating the patients exercises' performance are, despite of using only one sensing device to gather motion data. This is extremely important since the system should do this as if it were an expert that was near the patient while he performd the exercises. We carried out a qualitative study that consisted of checking the system's behaviour performing 16000 repetitions and counting the number of correct and erroneous repetitions well recognized. Results were quite satisfactory, achieving an accuracy average rate of almost 99% in both cases.

Secondly, we made a qualitative study that consisted of a survey about the users' perception of different issues related to the design and use of eFisioTrack. Participants answered 10 simple questions, giving answers that range from 0 to 5, being 5 the higher scoring. The scores were between 4.4 and 5, showing a high opinion of the system and the experience of using it.

The results of this evaluation make us conclude that the designed environment is appropriate and technically feasible.

Physiotherapists greatly appreciated the fact that our environment includes services that cover their needs throughout the whole rehabilitation process. Thanks to this we avoid the fact that the system was perceived as a disturb in their routine.

Another issue that our proposal solves is the gap between patients and experts. Physiotherapists found the interaction mechanisms that the system offers to them useful and suitable. These mechanisms help patients not to forget how to perform the exercises. Video and text descriptions of the rehabilitation sessions, together with a messaging mechanism, try to guide and help patient in the absence of the expert. The strengthening of the communication between physiotherapist and patient will benefit on the long course the results of the treatment, giving to the patient the feeling of a more centered-on-him healthcare process.

Regarding adherence to treatment, we consider that eFisioTrack can improve it since the amount of detailed data obtained from patients performance allows experts to be more precise in setting and updating the rehabilitation plans. On the other side, the system has many aspects to increase patients motivation to complete rehabilitation programmes. The use of innovative ways to perform the exercises, usually tedious for them, helps them to do it in a better mood. Besides, they know that the expert will immediately know if they are completing every session of the rehabilitation program, so in the next scheduled meeting they will not be able to lie to the expert.

## Figures and Tables

**Table 1:** Results of gestures recognition. Percentage accuracy values.

Performance	Exercises				Total accuracy average
	Flexion	Abduction	Leg extension	Adduction	
Erroneous	100	96.5	100	99.37	** 98.97**
Correct	99	98	100	98.25	** 98.81**

**Table 2:** Questionnaire and rating.

Questions	Ratings
(1) Do you consider that eFisioTrack properly covers all tasks related to the 1st examination of a patient?	5
(2) Do you consider that eFisioTrack properly covers all tasks related to an intermediate revision of a patient within a rehabilitation plan to check his evolution?	5
(3) Do you find the information related to patients exercises' performance that the system gathers adequate?	4.7
(4) The system offers to you enough mechanisms to interact with the patient?	5
(5) Does the use of eFisioTrack disturb your current workflow adding extra obstacles?	4.8
(6) Is the interaction with the system intuitive and friendly? (colors, icons, options placement, options menus, navigation through menus, alert and guiding messages, and so on)	4.4
(7) Do you consider that eFisioTrack's motion recognition is accurate enough?	4.8
(8) Do you consider that the use of eFisioTrack will help to increase patients' adherence to treatment?	4.7
(9) Do you consider that the use of eFisioTrack will help you to offer a better healthcare service?	4.5
(10) Do you consider that the use of eFisioTrack will help you to offer a more efficient healthcare service?	4.8

## References

[1] World Health Organization (2011). World report on disability. http://whqlibdoc.who.int/publications/2011/9789240685215_eng.pdf.

[2] Sabaté E (2003). Adherence to long-term therapies: evidence for action. http://www.who.int/entity/chp/knowledge/publications/adherence_full_report.pdf.

[3] Belza B, Topolski T, Kinne S, Patrick DL, Ramsey SD (2002). Does adherence make a difference? Results from a community-based aquatic exercise program. *Nursing Research*.

[4] Pisters MF, Veenhof C, Van Meeteren NLU (2007). Long-term effectiveness of exercise therapy in patients with osteoarthritis of the hip or knee: a systematic review. *Arthritis Care and Research*.

[5] Kolt GS, Brewer BW, Pizzari T, Schoo AMM, Garrett N (2007). The sport injury rehabilitation adherence scale: a reliable scale for use in clinical physiotherapy. *Physiotherapy*.

[6] Van Gool CH, Penninx BWJH, Kempen GIJM (2005). Effects of exercise adherence on physical function among overweight older adults with knee osteoarthritis. *Arthritis and Rheumatism*.

[7] Jack K, McLean SM, Moffett JK, Gardiner E (2010). Barriers to treatment adherence in physiotherapy outpatient clinics: a systematic review. *Manual Therapy*.

[8] Sniehotta FF, Scholz U, Schwarzer R (2005). Bridging the intention-behaviour gap: planning, self-efficacy, and action control in the adoption and maintenance of physical exercise. *Psychology & Health*.

[9] Milne MI, Hall CR, Forwell L (2005). Self-efficacy, imagery use, and adherence to rehabilitation by injured athletes. *Journal of Sport Rehabilitation*.

[10] Páez DG, Aparicio F, de Buenaga M, Padrón V (2012). Personalized health care system with virtual reality rehabilitation
and appropriate information for seniors. *Sensors*.

[11] Crosbie JH, Lennon S, McGoldrick MC, McNeill MD, McDonough SM (2012). Virtual reality in the rehabilitation of the arm after hemiplegic stroke: a randomized controlled pilot study. *Clinical Rehabilitation*.

[12] Henderson A, Korner-Bitensky N, Levin M (2007). Virtual reality in stroke rehabilitation: a systematic review of its effectiveness for upper limb motor recovery. *Topics in Stroke Rehabilitation*.

[13] Guberek R, Schneiberg S, McKinley P, Cosentino F, Levin MF, Sveistrup H Virtual reality as adjunctive therapy for upper limb rehabilitation in cerebral palsy.

[14] Cameirão MDS, Badia SBI, Duarte E, Verschure PFMJ (2011). Virtual reality based rehabilitation speeds up functional recovery of the upper extremities after stroke: a randomized controlled pilot study in the acute phase of stroke using the Rehabilitation Gaming System. *Restorative Neurology and Neuroscience*.

[15] Shi X-H, Wang H-B, Yuan L, Xu Z, Zhen H-W, Hou Z-G (2012). Design and analysis of a lower limb rehabilitation robot. *Advanced Materials Research*.

[16] Liu Y, Zhang L, Song Q, Shuang F, Ge Y (2012). Design and investigation on control system of a rehabilitation robot based on walking gait. *Advances in Computer, Communication, Control and Automation*.

[17] Zhou H, Hu H (2008). Human motion tracking for rehabilitation—a survey. *Biomedical Signal Processing and Control*.

[18] Khan AM, Lee Y-K, Lee S, Kim T-S (2010). Accelerometer's position independent physical activity recognition system for long-term activity monitoring in the elderly. *Medical & Biological Engineering & Computing*.

[19] Olivares A, Ramírez J, Górriz JMG, Olivares G, Damas M (2012). Detection of (in)activity periods in human body motion using inertial sensors: a comparative study. *Sensors*.

[20] Kratz S, Rohs M A $3 gesture recognizer: simple gesture recognition for devices equipped with 3D acceleration sensors.

[21] Tao Y, Hu H (2008). A novel sensing and data fusion system for 3-D arm motion tracking in telerehabilitation. *IEEE Transactions on Instrumentation and Measurement*.

[22] Schepers HM, Roetenberg D, Veltink PH (2010). Ambulatory human motion tracking by fusion of inertial and magnetic sensing with adaptive actuation. *Medical & Biological Engineering & Computing*.

[23] Banos O, Damas M, Pomares H, Rojas I (2012). On the use of sensor fusion to reduce the impact of rotational and additive noise in human activity recognition. *Sensors*.

[24] Lee JC (2008). Hacking the Nintendo Wii remote. *IEEE Pervasive Computing*.

[25] Schlömer T, Poppinga B, Henze N, Boll S Gesture recognition with a Wii controller.

[26] Leong TS, Lai J, Panza J, Pong P, Hong J Wii want to write: an accelerometer based gesture recognition
system.

[27] Loureiro R, Valentine D, Lamperd B, Collin C, Harwin W, Langdon PM, Clarkson PJ, Robinson P (2010). Gaming and social interactions in the rehabilitation
of brain injuries: a pilot study with the nintendo Wii console. *Designing Inclusive Interactions*.

[28] Tsekleves E, Skordoulis D, Paraskevopoulos I, Kilbride C Wii your health: a low-cost wireless system for home rehabilitation after stroke using Wii remotes with its expansions and blender.

[29] Fung V, Ho A, Shaffer J, Chung E, Gomez M (2012). Use of Nintendo Wii Fit in the rehabilitation of outpatients following total knee replacement: a preliminary randomised controlled trial. *Physiotherapy*.

[30] Zhang S, Hu H, Zhou H (2008). An interactive Internet-based system for tracking upper limb motion in home-based rehabilitation. *Medical & Biological Engineering & Computing*.

[31] Brennan DM, Lum PS, Uswatte G, Taub E, Gilmore BM, Barman J A telerehabilitation platform for home-based automated therapy of arm function.

[32] Brennan DM, Barker LM (2008). Human factors in the development and implementation of telerehabilitation systems. *Journal of Telemedicine and Telecare*.

[33] WiimoteLib .NET Managed Library for the Nintendo Wii Remote. http://www.brianpeek.com/page/wiimotelib.

[34] Sakoe H, Chiba S (1978). Dynamic programming algorithm optimization for spoken word recognition. *IEEE Transactions on Acoustics, Speech, and Signal Processing*.

[35] Raso I, Hervas R, Bravo J m-Physio: personalized accelerometer-based physical rehabilitation platform.

